# Unlocking Germanium Potential: Stabilization Strategies Through Wet Chemical Functionalization

**DOI:** 10.3390/ma17246285

**Published:** 2024-12-23

**Authors:** Alessia Arrigoni, Benedetta Maria Squeo, Mariacecilia Pasini

**Affiliations:** Istituto di Scienze e Tecnologie Chimiche “Giulio Natta”—SCITEC—CNR, Via Corti, 20132 Milan, Italy; alessia.arrigoni@scitec.cnr.it (A.A.); benedetta.squeo@scitec.cnr.it (B.M.S.)

**Keywords:** germanium, surface functionalization, surface passivation

## Abstract

Germanium (Ge) has long been recognized for its superior carrier mobility and narrower band gap compared to silicon, making it a promising candidate in microelectronics and optoelectronics. The recent demonstration of good biocompatibility, combined with the ability to selectively functionalize its surface, establishes the way for its use in biosensing and bioimaging. This review provides a comprehensive analysis of the most recent advancements in the wet chemical functionalization of germanium surfaces. Wet chemical methods, including Grignard reactions, hydrogermylation, self-assembled monolayers (SAMs) formation, and arylation, are discussed in terms of their stability, surface coverage, and potential for preventing reoxidation, one of the main limits for Ge practical use. Special emphasis is placed on the characterization techniques that have advanced our understanding of these functionalized surfaces, which are crucial in the immobilization of molecules/biomolecules for different technological applications. This review emphasizes the dual functionality of surface passivation techniques, demonstrating that, in addition to stabilizing and protecting the active material, surface functionalization can impart new functional properties for germanium-based biosensors and semiconductor devices.

## 1. Introduction

Germanium (Ge) has received significant attention in recent decades due to its promising electronic properties and potential applications in areas such as microelectronic and optoelectronic devices. As modern microelectronics technology advances, there is increasing interest in using Ge as a potential alternative to silicon. Ge offers indeed three times higher carrier mobility and a narrower band gap, making it an attractive option [1]. Additionally, Ge processing, including dopant activation, can be performed at lower temperatures compared to silicon. Indeed, Ge has a lower melting point (1210 K) than Si (1683 K) and its lattice parameters are similar to those of GaAs, allowing for potential integration of GaAs onto Ge-based chips [2].

**Scheme 1:**
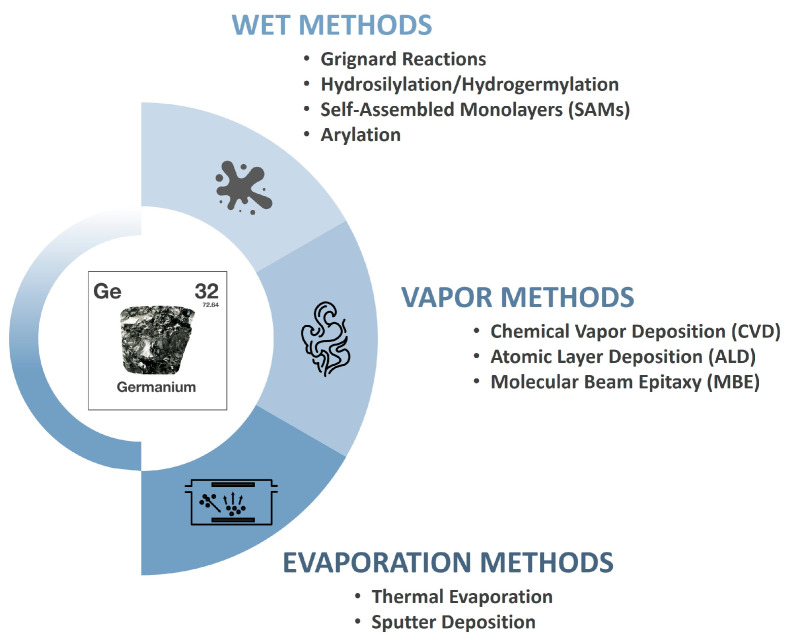
Schematical representation of the main techniques used for the functionalization and passivation of semiconductors like germanium (Ge) and silicon (Si).

To fully harness the potential of these materials and effectively tailor their optical and electronic properties as needed, it is essential to exploit the possibility of surface functionalization. The main techniques used for the functionalization and passivation of semiconductors like germanium (Ge) and silicon (Si) can be classified into three broad categories (Scheme 1):

Wet Chemical Methods (WM): These techniques utilize chemical reactions in a liquid phase to modify the surface of semiconductors [3]. Examples include hydrogermylation and Grignard reactions [4,5,6,7,8]. WM offer good surface coverage and flexibility in introducing specific functional groups, making them ideal for applications such as biosensors and bioimaging. Additionally, they often require simpler equipment compared to vapor deposition techniques, which demand vacuum systems, reaction chambers, and precise control units, contributing to higher energy consumption and costs. WM also allow the functionalization of large surface areas in a single process. Furthermore, organic solvents and reaction baths used in WM can often be purified and reused multiple times, minimizing waste and resource consumption. These characteristics make wet chemical methods a more accessible alternative, particularly for materials sensitive to high temperatures or those unsuitable for vapor deposition (e.g., with high molecular weights).

Vapor Deposition Methods [9]: Techniques like chemical vapor deposition (CVD) and atomic layer deposition (ALD) are based on depositing materials from a vapor phase onto a surface. These methods allow for highly uniform and controlled thin film growth, crucial for surface passivation and improving the electronic properties of semiconductors. They provide precise control over film thickness, making them essential for high-tech applications.

Evaporation Methods: This category includes techniques like thermal evaporation and sputtering [10]. The evaporation method, which is classified as a subset of vapor deposition techniques, involves heating a material to its vapor phase and physically depositing it onto a semiconductor surface under controlled conditions, typically in a high vacuum environment. With respect to CVD, which relies on chemical reactions in the vapor phase to form layers, evaporation is a purely physical process and does not involve chemical transformation of the material during deposition. This distinction is critical because evaporation, being a purely physical process, is simpler in terms of mechanism compared to techniques like CVD. However, it is primarily limited to materials that can transition to the vapor phase without undergoing decomposition, making it less versatile for certain types of compounds or applications. These methods are often used for metal or oxide deposition and are suitable for achieving stable functionalized surfaces. However, they typically require a vacuum environment and more advanced equipment.

WM offer several distinct advantages for the functionalization and passivation of semiconductors like germanium (Ge) and silicon (Si) compared to vapor methods and evaporation techniques [11,12,13,14,15,16]. In fact, WM, such as self-assembled monolayers (SAMs) and hydrosilylation, often achieve high surface coverage due to the intimate contact of the liquid phase with the semiconductor surface. This allows for uniform functionalization across the entire area, which is crucial for consistent device performance. Indeed, wet chemical techniques enable precise control over the surface chemistry and the types of functional groups introduced. By adjusting the concentration, reaction time, and temperature it is possible to tailor the properties of the surface to meet specific application needs. Moreover, WM typically require basic laboratory equipment, such as glassware, heating plates, and magnetic stirrers, which are significantly less complex compared to the advanced infrastructure necessary for vapor deposition techniques, such as high-vacuum systems, specialized reaction chambers, and precise control units for gas flow and temperature. This can lead to lower costs and easier scalability for industrial applications. Although WM require the use of organic solvents and reagents, the equipment needed for these processes is generally less expensive and less complex than that required for vapor of deposition and evaporation techniques, which demand complex infrastructure, including high-vacuum systems, specialized reaction chambers, and precise temperature and gas flow control. Additionally, WM are capable of functionalizing larger surface areas, making them more scalable for industrial use. The solvents employed in WM can often be purified and reused several times, further reducing operational costs. WM are also particularly beneficial for modifying materials sensitive to high temperatures, which would not be suitable for vapor deposition methods. This makes WM a valuable, accessible, and complementary alternative, especially for high molecular weight materials (which are extremely challenging to evaporate) or those requiring mild processing conditions. Additionally, the procedures often do not require high vacuum environments, making them more accessible for various laboratory settings. Another advantage is WM allow for a diverse range of chemical modifications. For example, organic molecules with varying functional groups can be used to achieve desired electronic, optical, and chemical properties. This versatility is particularly beneficial for applications in biosensing and bioimaging [17,18,19,20,21,22,23].

Ge has been shown to possess various biological functions, including antibacterial, antiviral, anti-inflammatory, anticancer, and antitumor activities, among others [24]. As a result, its significance as a material in biomedicine has grown in recent years, with promising application prospects. However, research on the biological effects and mechanisms of Ge remains limited, and a comprehensive systematic evaluation is still lacking [25]. In vitro cytotoxicity tests suggest that Ge-based materials and their dissolution products are biocompatible, highlighting their potential for use in temporary biomedical devices [26,27].

Additionally, functionalization through WM can help passivate dangling bonds on semiconductor surfaces, thus reducing electronic defects that can arise during the fabrication process. This leads to improved stability and enhanced electrical properties. It is worthy to mention WM, especially those that utilize biocompatible reagents, are advantageous for applications in biosensing and bioimaging. The ability to create functional surfaces that are compatible with biological molecules is crucial for the development of effective biosensors.

When considering the utilization of Ge in semiconductor applications, it is crucial to evaluate its inherent reactivity and instability.

**Scheme 2:**
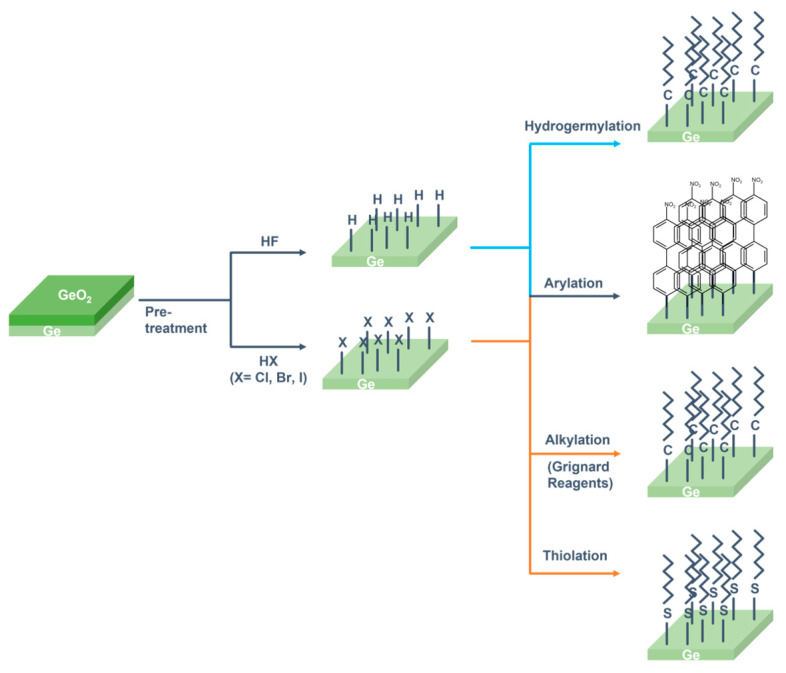
Schematic representation of the main WM routes covered by this review.

The wet chemical functionalization of Ge has evolved significantly over the decades, reflecting advancements in surface chemistry and the growing interest in Ge as a semiconductor material. Achieving stable and high-coverage surface functionalization is crucial not only for enhancing the stability of materials but also because it allows for easy modulation of optical and electronic properties [3,28,29]. A schematic representation of the main WM functionalization routes covered by this review is given in Scheme 2.

Nevertheless, prior to any organic functionalization, a key challenge in working with Ge lies in its surface preparation and passivation, which is more complex than that of silicon. The instability of Ge oxide has indeed hindered its widespread use in integrated circuits and several surface stability challenges need to be considered [30,31,32,33,34]. Native silicon oxide (SiO_2_) is a remarkably stable passivating layer, acts as a good electrical insulator, and forms an excellent interface with Si. In contrast to silicon, which forms a stable native oxide (SiO_2_) native Ge oxide (GeO_2_) behaves quite differently. Silicon forms a stable native oxide (SiO_2_) that can be readily grown thermally, whereas GeO_2_ is water soluble and forms a poor interface with Ge, which results in the facile removal of the oxide layer and a high-density of electronic defects [34,35,36,37]. Also, upon annealing, GeO_2_ transforms to GeO, which then desorbs from the surface at around 420 °C, making it unsuitable for device applications [36,38].

Ge surfaces are also prone to rapid reoxidation when exposed to ambient conditions, even after the oxide layer has been removed [1,39]. From this aspect arises the necessity of an effective surface passivation to enhance the stability and performance of Ge in electronic applications [39,40]. Passivation techniques, such as using hydrohalogenic acids (with HF yielding a H-terminated surface and HCl, HBr, and HI resulting in Cl-, Br-, and I-terminated surfaces, respectively), hydrogen peroxide, or organic acids, have been explored but exhibit limitations in terms of stability and effectiveness [8,41,42,43,44] H- and Cl-terminated Ge surfaces, in particular, quickly reoxidize, whereas sulfur- and alkyl-terminated surfaces have shown more resilience [37,39]. This highlights the ongoing challenges in stabilizing Ge surfaces, which remain a significant obstacle for the integration of Ge into high-performance electronic devices [37,38]. Stabilizing the Ge surface for effective chemical functionalization presents intrinsic challenges. However, WM remain advantageous over vapor deposition techniques in specific contexts, offering scalability, cost-effectiveness, and the ability to functionalize larger or irregularly shaped surfaces. The inherent challenges associated with Ge stabilization are addressed through optimized pretreatment protocols, which are critical regardless of the chosen functionalization approach.

The first approach to the functionalization of Ge surfaces was reported by Cullen et al. in 1962 [6], who utilized Grignard reagents to introduce ethyl groups onto chloride-terminated Ge (111) surfaces. This pioneering work demonstrated the potential for creating stable organic layers on Ge, although the method was limited by the harsh conditions required for the chlorination process, which could lead to surface roughness [36,37].

After this initial exploration, a renewed interest arose in the late 1990s, with the introduction of hydrogermylation reactions. Choi and Buriak were among the first to demonstrate this method on hydride-terminated Ge (100) surfaces; this method involves the direct attachment of carbon to Ge surfaces through the reaction of alkenes and alkynes with hydride-terminated Ge, forming covalently bonded organic monolayers [5]. The hydrogermylation process can be achieved through various activation methods, including Lewis acid mediation and thermal activation, providing flexibility in the functionalization process [36].

In the early 2000s, a growing interest arose in using alkanethiols to create SAMs for functionalizing Ge surfaces. This method involves dipping hydrogen-terminated or halogen-terminated Ge into alkanethiol solutions, leading to the formation of stable SAMs. Studies have shown that the stability of these monolayers can be affected by factors such as the length of the alkanethiol chain and the crystallographic orientation of the Ge substrate [37]. The stability of these thiol-based SAMs was found to be superior to that of Grignard-alkylated surfaces, particularly in terms of oxidation resistance. This was a significant finding, as it highlighted the potential for thiol chemistry to enhance the ambient stability of Ge surfaces, which had been a persistent challenge in the field.

In more recent years, the focus has shifted toward the use of diazonium salts for functionalization, which offers a rapid and efficient means of grafting organic layers onto Ge surfaces at room temperature [45,46]. This method not only simplifies the functionalization process but also enhances the stability of the resulting organic films, making it a promising avenue for future research and applications in biosensing and other fields.

The present review aims to provide an overview of the main wet techniques used for Ge functionalization. In the first section, the evolution of the concept of functionalization is highlighted, showing how it has shifted from a simple surface stabilization strategy to a method for modulating surface properties. A brief paragraph is also dedicated to substrate preparation techniques, maintaining the chronological order of their development. This is an essential preliminary step before functionalization. The review then goes into detail about the various wet functionalization methods, with a focus on the characterization techniques employed for analyzing these surfaces. Here, we decided to focus on wet-chemical functionalization techniques applied to bulk Ge surfaces. While recent studies on germanium-based nanomaterials, including quantum dots (Ge-QDs), nanowires, and other low-dimensional structures, have demonstrated intriguing applications and functionalization capabilities, they are not the primary focus of this manuscript [47,48,49,50,51,52]. These materials often exhibit distinct chemical and physical behaviors compared to bulk surfaces due to their unique size-dependent properties and high surface-to-volume ratios. Nevertheless, we have included select findings on the functionalization of germanium nanowires, as they provide an interesting comparative perspective to bulk Ge surfaces [4,8,46,53]. Finally, perspectives on the developments in Ge-surface chemistry are illustrated.

## 2. Importance of Surface Functionalization of Germanium

As previously mentioned, the functionalization step is crucial in the development of Ge-based systems as it is capable of enhancing the stability of the Ge surfaces and to impart new and specific functionalities to the surface. Indeed, grafting molecules such as alkanethiols or other groups can create a protective layer that can resist oxidation [8,54]. Functionalization is also important in the enhancement of electronic properties of Ge since, by modifying the surface chemistry, functionalization can make it more suitable for high-performance applications in electronics, such as field-effect transistors (FETs) and photodetectors [8,54]. Also, functionalized Ge surfaces can be designed to allow for the attachment of biomolecules, making them useful for biosensor applications. For instance, calixarene-based functionalization can prevent nonspecific adsorption while allowing for specific binding of proteins [23].

The absence of a stable native oxide on Ge has historically been considered a disadvantage. However, the formation of Ge-C or Ge-S bonds offers a promising alternative that has proven to be convenient for further use in Ge-based devices [55]. This will facilitate the integration of Ge into various technological applications by customizing its properties and enhancing stability.

**Figure 1:**
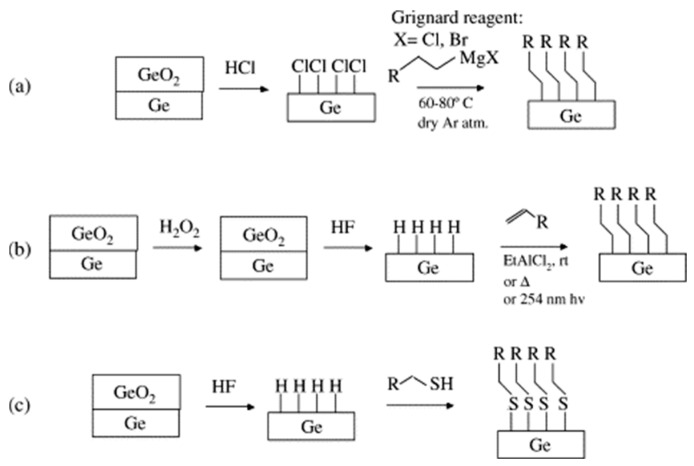
Alkylation routes of Ge surfaces (**a**) through a Grignard reaction, (**b**) through hydrogermylation, and (**c**) through reaction with thiols. Reproduced with permission from [39] by Elsevier.

A number of routes to nominally stable termination/passivation layers of Ge have been reported and are explored in the following sections of this review. These are schematized in Figure 1. For example, Cl-terminated Ge surfaces can be further functionalized by Grignard reagent to attach alkyl monolayers. H-terminated surfaces can follow a hydrogermylation reaction to alkylate Ge substrates, or grafting of Ge surface can also proceed through self-assembly of alkanethiols.

Before delving into the discussion of wet functionalization techniques, it is important to clarify the difference between passivation and functionalization steps. In surface chemistry, particularly in the context of semiconductor materials such as Ge and silicon, functionalization and passivation are two separate processes. Here are the key differences:

- *Passivation* refers to the step aimed at making a surface less reactive or inert. Passivation typically involves the formation of a protective layer that prevents further chemical reactions, such as oxidation, thereby stabilizing the surface [1]. The main goal is to protect the surface from environmental factors that could lead to degradation, such as oxidation or contamination. This is crucial for maintaining the integrity and performance of semiconductor devices [1]. For example, a layer of sulfur or halides can create a stable, inert surface that resists oxidation and maintains the desired electronic properties of the semiconductor [1].
- *Functionalization* refers instead to the process of chemically modifying a surface by attaching specific functional groups or molecules to it. The goal in this case is to impart new chemical properties or functionalities to the surface, which can enhance its reactivity, selectivity, or compatibility with other materials [56]. The primary aim is to introduce specific chemical functionalities that can interact with other molecules or materials. This can be used for applications such as biosensing, drug delivery, or improving electronic properties [57,58]. For example, by attaching alkanethiols to a surface can create a self-assembled monolayer that can interact with biomolecules or other chemical species [53,59].

In short, both processes involve the alteration of a surface, but functionalization entails adding specific chemical functionalities, while passivation focuses on protecting the surface from undesired reactions. Generally, passivation precedes functionalization in the context of semiconductor surfaces, including Ge, and is essential for preparing the surface for subsequent functionalization. For instance, in the case of Ge, passivation methods such as hydrogen termination or halogen termination are often used to stabilize the surface before applying functionalization techniques like SAMs or other chemical modifications [30,34]. This sequence ensures that the functionalization process can occur on a clean and stable surface, enhancing the effectiveness and durability of the functionalized layer.

## 3. Pretreatments of Germanium Substrates

The removal of the native oxide from Ge surfaces is a critical preparatory step for ensuring high-quality functionalization. Over the years, various techniques have been developed, but our focus here is specifically on wet chemical methods. These approaches are particularly effective in creating H- or halogen-terminated Ge surfaces, which enhance stability and serve as an ideal foundation for further functionalization.

One of the most commonly used methods for oxide removal is hydrofluoric acid (HF) treatment. This method effectively etches away the native Ge oxide (GeO_2_) and results in hydrogen-terminated surfaces (Ge-H). While from a thermodynamic point of view, an F passivated surface is more stable, studies on Si have been demonstrated that kinetics plays a more important role in the surface hydrogen passivation [60,61]. These studies refer to Si; however, since Ge has an electronegativity very close to that of Si, similar arguments can be applied. During the etching process, H+ etches the oxide and passivates the Ge surfaces, leaving an H-terminated surface [44]. A typical procedure involves etching the Ge surface in a 10% HF solution for a specified duration (e.g., 1–10 min) [5]. However, studies indicate that while HF treatment can effectively remove the oxide layer [5,62], it does not completely eliminate the suboxide layer (GeO_x_) that may remain on the surface [39]. The stability of the hydrogen-terminated surface is also limited to several minutes, as it tends to reoxidize quickly upon exposure to ambient conditions, water vapors or a mixture of oxygen and water vapor [8,43,44,63,64]. For instance, after treatment with 20% HF, the H-terminated surface can reoxidize within minutes, making it less suitable for long-term applications [37].

Passivation of the Ge surface by using hydrochloric acid (HCl), hydrobromic acid (HBr), or hydroiodic acid (HI), in contrast, has been shown to efficiently remove the surface oxide by terminating the surface with the associated halogen atom simultaneously [38,65,66]. HCl, HBr, and HI treatments (commonly used in concentrations ranging from 5% to 20% in aqueous solutions, as reported in the literature) not only remove the oxide, but also provide a Cl (or Br, I)-terminated surface that exhibits greater resistance to reoxidation compared to hydrogen-terminated surfaces. Studies on ambient stability show that the surfaces terminated with Cl- or Br- remain stable against reoxidation for 10 min and 6 h after exposure to ambient atmosphere, respectively [8,62,64,67,68].

Additionally, research has demonstrated that treating with hydrochloric acid (HCl) produces a smoother surface compared to treatment with hydrofluoric acid (HF), which can cause roughness by breaking the back-bonds in Ge [8]. Achieving an acid-treated surface that is as smooth as possible is essential for effective passivation, facilitating subsequent organic functionalization and enabling its application in nanoscale electronics. Hydrohalogenic acids, such as HF, HCl, and HBr, have been reported to effectively remove oxides from Ge surfaces, though with varying degrees. Lefèvre and colleagues demonstrated that treatments combining these acids with H_2_O_2_ in a cyclic oxidation–rinsing–etching process result in notably different surface roughness levels, depending on the acid used [45].

**Figure 2:**
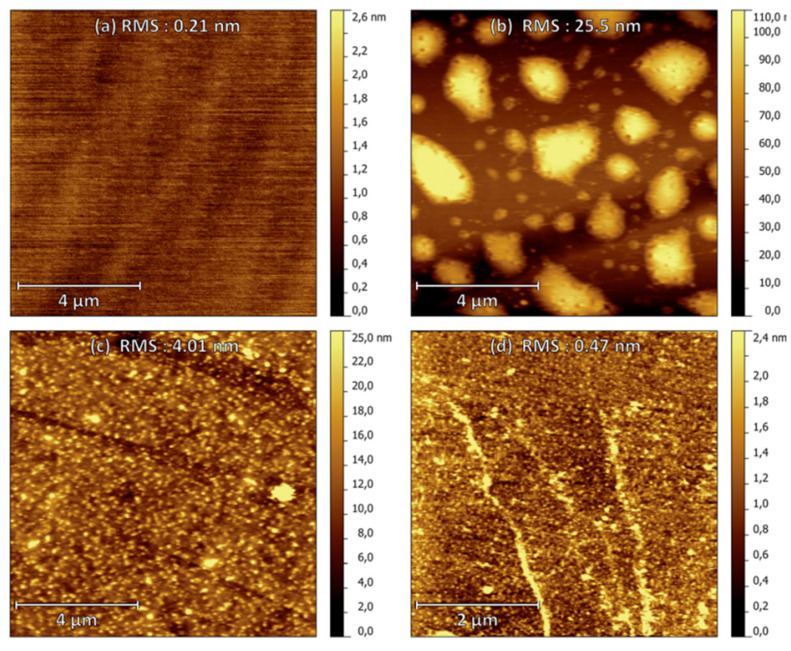
AFM images of as-received (**a**) Ge; (**b**) piranha-treated Ge, showing extensive damage on the surface; (**c**) H_2_O_2_ (30%)/HBr (10%) cyclic-procedure-treated Ge, showing a smoother surface; and (**d**) H_2_O_2_ (30%)/HCl (10%) cyclic-procedure-treated Ge showing the smoothest surface obtained. The differences in the vertical scales are directly related with the very large variations in roughness among the four images. Reproduced with permission from [45] by Royal Society of Chemistry.

Among the cyclic procedures involving H_2_O_2_-hydrohalogenic acids, the treatment with HCl yielded the best results in terms of final surface roughness, while treatments with HBr and HF were found to be more harmful to the surface, as reported in Figure 2. Exposing Ge surfaces to more aggressive treatments such as a 30 s piranha solution (sulfuric acid (H₂SO₄) and hydrogen peroxide (H₂O₂), commonly in a ratio of 3:1 or 4:1 by volume) significantly damaged the surfaces, resulting in a roughness of 25.5 nm with noticeable irregularities, whereas the pristine Ge surfaces had a roughness of around 0.3 nm.

Other reported wet chemistry treatments include the use of hydrogen peroxide (H_2_O_2_, 30% for 10 s) combined with deionized water treatment to remove GeO_2_ [5]. Additionally, NH_4_OH-based (28%) treatments are employed to minimize carbon contamination [69], while (NH_4_)_2_S treatments result in S-terminated surfaces [39]. A cyclic cleaning process using H_2_O_2_ and HF or HCl is also effective for oxide removal and surface preparation for subsequent functionalization [70]. In this case, the last step should involve immersion in HF or HCl, to leave an H- or Cl-terminated surface, ready for the subsequent functionalization step.

Another method that has recently gained attention is the use of citric acid for oxide removal. Citric acid (0.24 M–2.4 M), a natural organic acid obtained through sustainable processes like biofermentation, is significantly less aggressive than inorganic acids. Additionally, citric acid offers several environmental advantages: it is low in toxicity, easy to handle, and poses no disposal challenges, aligning well with green chemistry principles. It effectively removes both GeO₂ and GeOx and can passivate the surface by chelating Ge atoms. This results in a surface that is comparable in stability to that achieved with HCl treatment [41,42]. However, it is crucial to note that residual citric acid can inhibit the subsequent deposition of alkanethiol layers, as it may block surface sites necessary for functionalization [41]. This indicates that while citric acid is effective in oxide removal, additional steps may be required to ensure a clean surface for further functionalization.

Nevertheless, after relatively short periods of ambient exposure, the oxide-free Ge surfaces mentioned so far display a significant reoxidation. For this reason, postfunctionalization of these Ge surfaces is necessary to stabilize the oxide-free surface as well as impart further functionalities for practical applications.

However, despite the effectiveness of these treatments, acids like HF and HCl, commonly employed for oxide removal and surface activation, are hazardous and must be handled with extreme care. Their toxicity is a critical consideration, although they are frequently used in controlled environments with low concentrations (typically between 5% and 10% in aqueous solutions).

Mitigation strategies to address the toxicity of these acids include optimizing reaction protocols (e.g., using alternative reagents with lower toxicity, recycling solvents, and implementing proper waste management systems). Efforts to replace HF or HCl with milder etchants, such as citric acid as previously reported, have shown promise in reducing toxicity while maintaining surface quality. Additionally, optimizing reaction conditions to reduce the concentration of hazardous reagents and ensuring that they are highly diluted in water helps minimize potential risks. Proper waste management systems, including solvent recycling and neutralization of acid wastes, are crucial to reduce the environmental impact of these chemicals. Through these measures, it is possible to improve safety and sustainability in the use of surface pretreatment chemicals without compromising the quality of the Ge surfaces.

## 4. Wet Chemical Functionalization Techniques and Comparative Analysis

Wet chemical functionalization of Ge involves various methods aimed at modifying its surface properties through chemical reactions in liquid solutions.

As previously mentioned, the earliest method for functionalizing Ge surfaces was reported in 1962 by Cullen et al. [37], who utilized Grignard reagents to introduce ethyl groups onto chloride-terminated Ge surfaces. This pioneering work demonstrated the potential for creating stable organic layers, which can significantly improve the chemical and electrical properties of Ge [37]. Following this, the field saw the introduction of hydrogermylation, a method that allows for the direct attachment of carbon to Ge surfaces. Choi and Buriak first demonstrated this technique using hydride-terminated Ge (100) surfaces, employing various activation methods such as Lewis acid mediation, UV light, and thermal activation [5]. Organic solvents commonly used in wet processes can have a considerable environmental footprint. However, many of these solvents are recoverable, can be purified, and reused multiple times, greatly minimizing their environmental effects.

Table 1 summarizes the pretreatment, the functionalization method, and the characterization techniques for different pure Ge substrates reported in this review. The first column of Table 1 summarizes the types of substrates tested for wet functionalization, as reported in the literature. Although a direct comparison between Ge (100) and Ge (111) is not available, Ge (100) has been reported to be more reactive toward vapor-phase functionalization [34]. This increased reactivity is attributed to its double bond-like character, resulting from the weak π bond between the atoms, as well as the zwitterionic-like nature of Ge dimers in the lattice structure. These characteristics enhance both cycloaddition and nucleophilic/electrophilic reactions in the gas phase. However, this difference in reactivity has not been observed for wet functionalization.

### 4.1. Alkylation (Ge-C)

The alkylation of Ge has been a significant area of research, mostly on the early stages of research concerning the functionalization of Ge substrates. This process involves the reaction of Grignard reagents with Ge-Cl surfaces using different alkyl chain lengths and, eventually, functional terminals.

Surfaces of Ge passivated with alkyl chains exhibit significantly greater stability compared to surfaces passivated with hydrogen or halogen atoms (Cl, Br, I). This is due to the strong Ge-C bond (460 kJ/mol) and the formation of a hydrophobic layer that blocks the access of oxidizing species to the Ge surface [8].

The pioneering work in this field dates back to 1962 when Cullen and coworkers introduced the concept of stabilizing Ge surfaces through ethylation using Grignard reagents. The treatment of a Ge (111) surface with gaseous HCl resulted in the creation of a chloride-terminated surface. Subsequent treatment with ethyl Grignard led to the successful formation of stable ethyl groups on the modified surface. The ethylation process involved the formation of Ge-C bonds in a one-to-one ratio with the surface atoms. Although this method demonstrated the potential to create a chemically inert and electronically insulating layer, the stability of the resulting surface was found to be limited at elevated temperatures, exhibiting hydrophobic behavior and good stability in both atmosphere and aqueous solutions [6]. Following this initial study, in the late 1990s, He et al. advanced the alkylation process by employing milder chlorination conditions using aqueous HCl, which minimized surface roughness compared to previous methods [7]. This approach facilitated the effective alkylation of Ge surfaces using Grignard reagents. Grignard reagents, such as CH_3_(CH_2_)_n_MgX (where n = 1, 2, 9, 13, 14, 18 and X = Cl and Br) and R(CH_2_)_2_MgX (R = C_6_H_5_ and CH=CH_2_), react with Cl-terminated Ge (111) surfaces at 60 to 80 °C under an inert Ar atmosphere. The concentration of Grignard reagents usually prepared in ether solvents varies between 0.5 M and 1 M. The resulting alkyl monolayer forms a covalent bond with Ge; it requires a long time, 7 days, to form an octadecyl mono layer. However, this method has been reported to be effective for the functionalization of both bulk [6,7] and nanowire [8] surfaces of Ge.

In terms of comparative stability, it has been reported that while alkylation via Grignard reagents produces stable functionalized surfaces, the presence of residual halogen species on halogen-terminated surfaces can lead to increased susceptibility to oxidation [8]. The primary drawback of alkyl functionalization is the different reactivity depending on the length of the alkyl chains. Typically, longer alkyl chains result in higher resistance to oxidation. However, long alkyl chains require longer reaction times, leading to lower reactivity toward Ge-C bond formation that can result in poorly packed molecular films, with some Ge with unreacted halogen species that are more prone to oxidation.

In summary, alkylation by Grignard reagents has been a common approach for functionalizing Ge that can yield stable organic layers [45], but the reaction often requires elevated temperatures, extended reaction times (from six hours to several days), and, as previously reported, the steric hindrance associated with longer alkyl chains can hinder the reaction efficiency [8].

### 4.2. Hydrogermylation (Ge-C)

One of the most extensively studied wet chemical functionalization methods for silicon is hydrosilylation. In this reaction, an unsaturated C-C bond is added across a Si-H bond. Similarly, the corresponding reaction on Ge, known as hydrogermylation, holds great potential for directly linking carbon to Ge surfaces [55].

**Figure 3:**
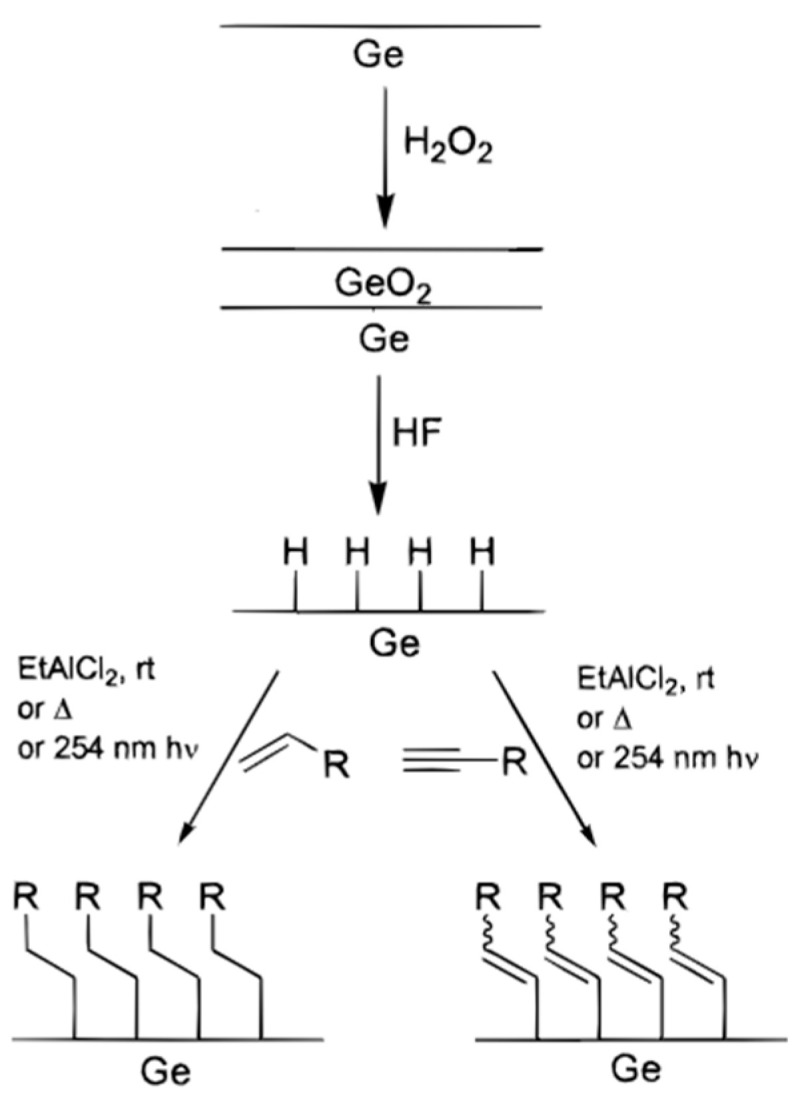
Scheme of the hydrogermylation reaction with alkenes and alkynes followed in ref. [31]. Reproduced with permission from [5] by American Chemical Society.

In 2000, Choi and Buriak conducted pioneering work on hydrogermylation, demonstrating the reaction of alkenes and alkynes with hydride-terminated Ge (100) surfaces [5]. Based on the stability of these monolayers and FTIR studies of the results of alkyne hydrogermylation, they conclude that the reactions result in the formation of Ge-C bonds. They achieved hydrogermylation using three different approaches: Lewis acid mediation; UV light (irradiation was carried out with a mercury vapor lamp at wavelengths of 350 and 254 nm for 1–2 h under an inert atmosphere (N_2_)); and thermal activation (by placing a H-terminated Ge substrate in an alkyne or olefin solution (3–100% *v*/*v* in mesitylene)—the solution was refluxed by placing the flask in an oil bath at 200–220 °C for 2 h under an inert atmosphere (Ar)). Lewis acid-mediated hydrogermylation using 1.0 M hexane solution of EtAlCl_2_ allows for the complete reaction with alkynes in 1 h and alkenes in 12 h at room temperature, producing alkenyl and alkyl monolayers, respectively. The reaction path is highlighted in Figure 3. This method offers potential advantages over Grignard reactions, which require between 6 h and 7 days at elevated temperatures for complete reactions [5].

The stability of the resulting monolayers was significant; nonetheless, the stability is highly dependent on the hydrogermylation route. Stability has been tested by following ATR-FTIR spectra in the alkyl CH stretching region. Results show that there is no change in the IR alkyl peak intensities for the thermally induced monolayer upon immersion in boiling water for 20 min followed by immersion in boiling chloroform for other 20 min. However, a similar treatment for the Lewis acid-mediated monolayer results in a 20% decrease in the IR intensity when immersed in boiling water and a 30% decrease when sonicated in chloroform [5].

Later, Hanrath and Korgel showed that the hydrogermylation reaction is also applicable to H-terminated Ge NWs [71]. They employed a thermally activated hydrogermylation reaction to passivate Ge NWs. It is noteworthy that they were able to achieve chemically stable alkyl-passivated Ge surfaces in situ using a single one-component injection step, without halogenating the surface. This process minimizes exposure of the NWs to air prior to surface passivation. The passivated NWs showed excellent resistance, monitored by X-ray photoelectron spectroscopy (XPS), for several conditions. Hydrogermylated NWs subjected to various oxidizing and corrosive conditions showed no observable oxidation signal after exposure to air for 1 week and remained unchanged even up to one month of air exposure. NWs also showed high resistance when exposed to water and 2.5 M sulfuric acid, with no signals of surface oxidation. This can be explained by the hydrophobicity of the passivating layer, which prevents penetration of water and aqueous sulfuric acid and prevents surface oxidation. Stronger oxidizing agents, such as hydrogen peroxide and sulfuric acid in dioxane, did corrode the NWs [4].

In summary, hydrogermylation has been extensively studied for its ability to create stable, covalently bonded organic layers on Ge surfaces. Choi and Buriak [5] demonstrated that hydrogermylation of alkenes and alkynes could be achieved under mild conditions, resulting in well-ordered monolayers. This method offers significant advantages in terms of the stability and robustness of the resulting layers, as alkyl-terminated surfaces exhibit excellent resistance to oxidation and degradation. However, the requirement for specific reaction conditions, such as the presence of Lewis acids or UV light, can complicate the process. However, the hydrogermylation reaction may not be compatible with all functional groups, limiting the versatility of the resulting surfaces.

### 4.3. Thiolation (Ge-S)

**Figure 4:**
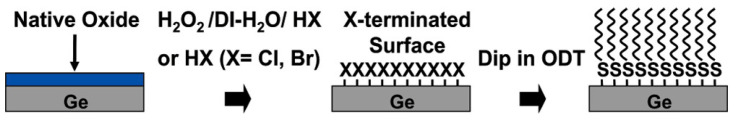
Schematic illustration of the formation of 1-alkanethiolate self-assembled monolayers on a Ge substrate using halide passivation followed by thiolation. Reproduced with permission from [37] by American Chemical Society.

In contrast to Grignard and hydrogermylation reactions, which create Ge-C bonds, the thiolation process forms a Ge-S bond through functionalization with alkanethiols. A schematic illustration of the thiolation process is given in Figure 4. The thiolation of Ge has been a focal point in surface chemistry. Compared to alkylated surfaces, thiolation of halogenated Ge surfaces is reported to provide better stability against oxidation [8] due to the better packing of the chains constituting the SAM, with only few sites exposed to oxidation. As reported by Collins et al. [8], the absence of halogen species in the XPS survey spectra after thiol functionalization indicates that alkanethiols are more effective in replacing surface halogen species compared to alkyl Grignard reagents. This effect is likely a result of the increased steric effects experienced by Grignard reagents due to solvent coordination. This coordination hinders their ability to access the halogenated species on the surface, leading to the presence of unreacted residual halogen species that were detected by XPS analysis.

The first study exploring alkanethiol functionalization was conducted in 2001 [72] and inspired by the results of Anderson et al. reporting that (NH_4_)_2_S can react with Ge in an aqueous solution [75]. These considerations led to the correct hypothesis that alkanethiols could react with Ge surfaces, producing an alkyl monolayer able to passivate the surface [39]. In 2001, Han et al. [72] demonstrated that it is possible to create an alkanethiol layer by immersion in an alkanethiol solution (10^−3^ M in 2-propanol) on an HF-treated Ge surface. The resulting surface is hydrophobic (as shown by contact angle measurements), confirming the alkylation of the surface. The monolayer formed is thermally stable at temperatures up to 450 K and air-stable for up to 12 h, indicating that thiols are covalently bonded to Ge. In 2009, Ardalan et al. [37] reported the self-assembly of long chain 1-alkanethiolates on halide terminated (Cl- and Br-) Ge substrates by immersion in 1-octadecanethiolate 10^−4^–10^−1^ M solutions in 2-propanol. IR investigation showed the formation of highly ordered, crystalline-like 1-octadecanethiolate SAMs at the Ge (100) surface. Water contact angles (WCAs) and film thickness (FTs) results indicate that SAM formation depends on the concentration of the alkanethiol, the solvent used, the crystallographic orientation of the substrate, and the type of surface passivation. The stability of the SAM has also been tested by monitoring changes in WCA and FT, highlighting a promising resistance of up to two months when the sample is conserved under dark conditions. However, when exposed to light, it degrades in a few days. Interestingly, it has been shown that a rehalogenation/rethiolation treatment can be performed to restore the SAM.

In 2010, Ardalan et al. [38], to better clarify the details of bonding structures, utilized synchrotron radiation photoemission spectroscopy (SR-PES) in combination with DFT calculations to demonstrate that thiolated Ge (100) and (111) surfaces consist primarily of monothiolates and dithiolates, along with unbound thiol and atomic sulfur, at room temperature. SAMs have been prepared by immersing the previously halide-passivated substrate in a 0.1 M solution of 1-octadecanethiol (ODT) in 2-propanol for 48 or 72 h. They also performed thermal annealing studies, showing that Ge-thiolates are thermally stable up to 150 °C and that most of the surface thiolates are converted to sulfide and carbide via S-C bond scission upon annealing at 350 °C.

The formation of self-assembled monolayers through alkanethiol can also be carried out on Ge NWs. Collins et al. [8] showed that NWs functionalized with alkanethiol have greater resistance against reoxidation compared to those functionalized with alkyl groups, using the Grignard reaction, when exposed to ambient conditions for 1 week.

As previously reported, the initial removal of native oxide and surface passivation are typically essential steps because the presence of native Ge oxide generally prevents the direct self-assembly of alkanethiols on the Ge surface. However, in 2011, Hohman et al. [30] presented an intriguing study in which, by adjusting the reaction conditions, they achieved the spontaneous formation of monolayers on untreated Ge surfaces. The alkanethiol was dissolved in a mixture of 1:1 water and ethanol obtaining a 0.5 mM solution, creating an environment where both the adsorbate and the oxide were sufficiently soluble. This approach facilitated the formation of a SAM in a single step. Temperature also played a crucial role in the results: when the deposition occurred at room temperature, the maximum coverage achieved was 50%. In contrast, increasing the temperature to 60 °C allowed for full coverage of the surface.

Through careful optimization of factors such as solvents (10 mM of 1-dodecanethiol are dissolved in a mixture solvent (water/ethanol, *v*/*v* = 1:1)), reaction time (24 h), and temperature (∼25 °C), Cai et al. [73] developed a 1-dodecanethiol SAM on Ge surface that remained stable for over 10 days, even in water conditions. This is an impressive improvement over the previously reported limit of 3 days [42]. Recently, Arrigoni et al. [76], by using fluorescent thiol-based molecules as probes [77], demonstrated that a more effective functionalization can be achieved during thiolation processes by using dry reagents and an inert atmosphere.

**Figure 5:**
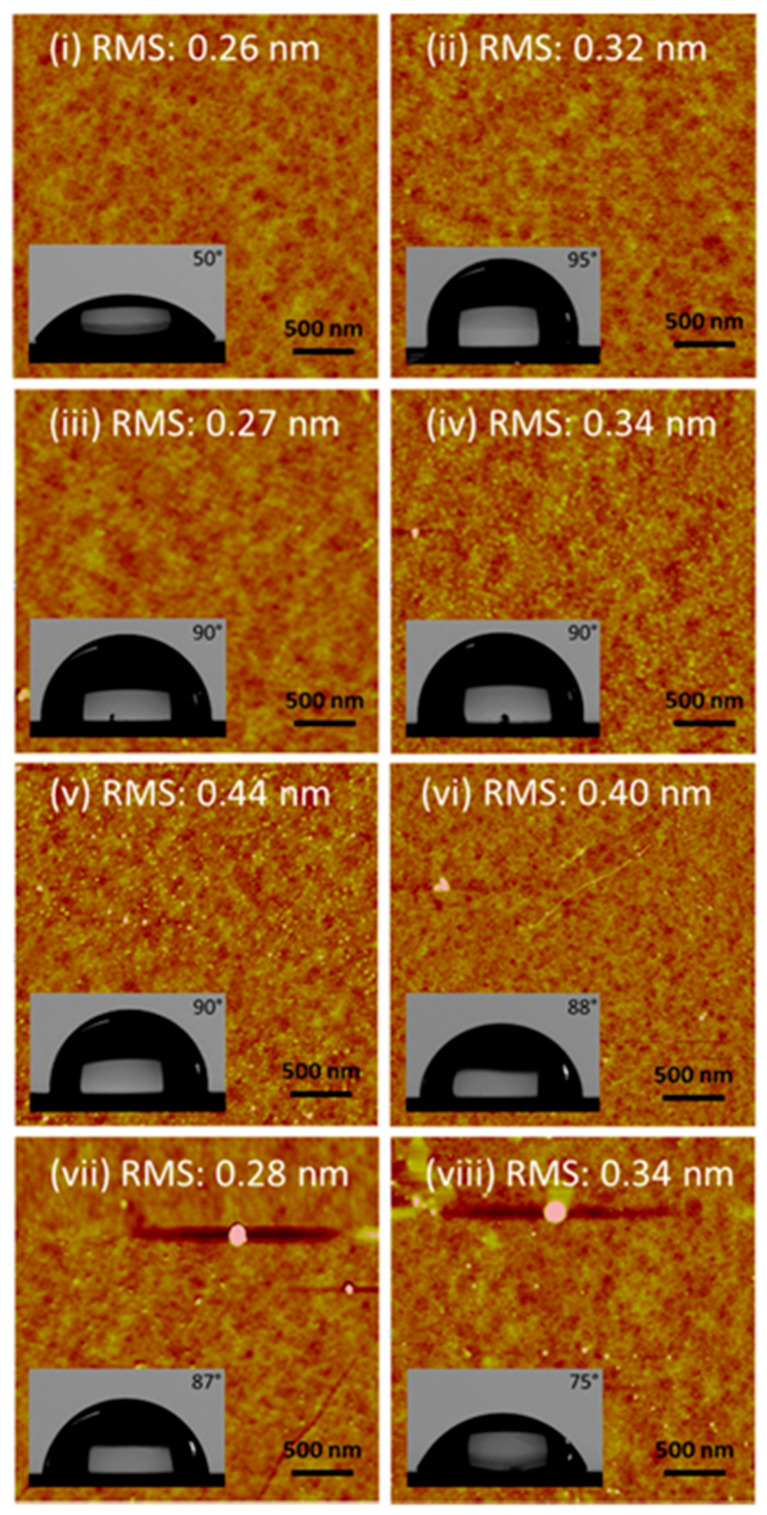
WCA and AFM data for (**i**) as-received Ge; (**ii**) HT-passivated Ge (**iii**,**iv**); HT-passivated Ge with 24 and 168 h exposure to a low humidity environment, respectively, (**v**,**vi**); HT-passivated Ge with 24 and 168 h exposure to an ambient humidity environment, respectively, (**vii**,**viii**); and HT-passivated Ge with 24 and 168 h exposure to a high humidity environment, respectively. Reproduced with permission from [1] by Elsevier.

The stability of thiolated Ge surfaces is critical for applications in electronics, where durability under environmental conditions is essential. Numerous studies have sought to understand the factors contributing to the high oxidation resistance of thiol-functionalized surfaces. Garvey et al. found that alkanethiol-passivated Ge (100) surfaces maintained their integrity in low-humidity environments, demonstrating significantly enhanced longevity compared to untreated surfaces [1]. The growth of native GeO_2_ over time was tracked using XPS to understand the impact of water vapor on SAM degradation and Ge surface oxidation. The XPS results showed that higher humidity led to increased oxidation rates of the thiol-Ge surface. WCA analysis supported these findings, showing a greater decrease in contact angle for Ge samples exposed to high humidity compared to those in lower humidity environments, as reported in Figure 5. WCA analysis was performed on each sample immediately after the passivation procedure and after 24 and 168 h in their respective environments. The native oxide film on the untreated Ge surface promotes favorable interaction with water, creating a hydrophilic surface with a contact angle between the water droplet, Ge, and air at about 50°. In contrast, replacing the oxide with a methyl-terminated monolayer (a hydrophobic functionalization) reduces interaction with polar water molecules, leading to decreased surface wetting and an increased contact angle at about 95° between water, Ge, and air (Figure 5). The AFM images in Figure 5 show, together with contact angle, the effect of different treatments on the surface roughness of the Ge. A pristine surface with a roughness RMS value of 0.26 nm was observed for the degreased Ge sample (refer to Figure 5i). An increase in surface roughness was noted for all other samples, and some spots were observed on the samples exposed to high relative humidity, possibly indicating oxide formations on the Ge surface at point defects.

Experimental and modeling results indicate that controlling environmental humidity can enhance the longevity of the thiol-passivated Ge surface and its resistance to oxidation. Alternatively, stability can be improved by optimizing the self-assembled monolayer itself—such as increasing tail group hydrophobicity with a fluorinated group, enhancing chain alignment and packing density, or reducing defect density [1].

**Figure 6:**
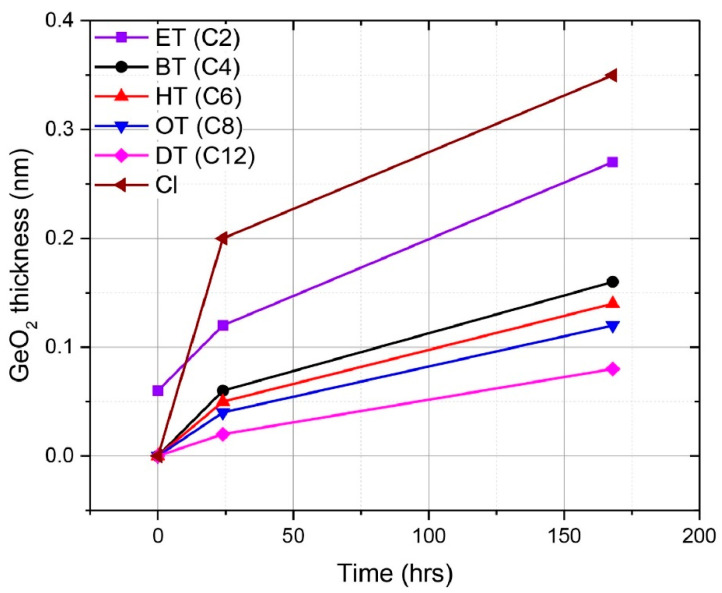
GeO_2_ growth over 24 and 168 h for Ge passivated by ET (purple), BT (black), HT (red), OT (blue), DT (pink), Cl (brown). Reproduced with permission from [59] by Elsevier.

The comparative stability of SAM on Ge surfaces was further examined by Garvey et al. (2020), who investigated the effect of alkanethiol chain length on the oxidation resistance [59]. Five thiol molecules (ethanethiol (ET), 1-butanethiol (BT), 1-hexanethiol (HT), 1-octanethiol (OT), 1-dodecanethiol (DT)) with increasing chain length were selected and used to passivate Ge. These substrates were studied in terms of reoxidation upon exposure to ambient conditions. In Figure 6, the GeO_2_ thickness, calculated by XPS measurements, is plotted against time, revealing that SAMs of thiol molecules with longer chains are more effective at inhibiting the oxidation of Ge compared to SAMs with shorter chains when exposed to ambient conditions for 168 h. Over this time period, GeO_2_ grows by 0.08 nm on DT-passivated Ge, while it grows by 0.12 nm, 0.14 nm, and 0.16 nm on OT-, HT-, and BT-passivated Ge, respectively.

This study showed that longer-chain alkanethiols provided increased protection against oxidation. Stability and coverage appear to be influenced by the tilt and orientation of the thiolate chains on the Ge surface. Longer carbon chains more effectively prevent Ge oxidation due to the van der Waals (vdW) forces between the carbon backbones of the adjacent thiol molecules. The overall vdW interaction increases as the chain length increases, thereby improving the effectiveness of the passivation.

Lately, there has been a growing interest in hybrid semiconductor biosensors based on Ge substrates that can electrically detect various biological targets. In this context, effectively immobilizing organic biological molecules on traditional semiconductor materials is crucial. In 2014, Cai and colleagues [74] introduced a simple yet effective method to immobilize biomolecules on the surface of Ge functionalized with 11-mercaptoundecanoic acid (11-MUA) (25 mM in 2-propanol) SAMs. After activating its carboxyl groups, this SAM successfully immobilized a green fluorescent protein (GFP, FTIC-tagged goat antimouse IgG antibodies). Contact angle and AFM measurements confirmed the stability and uniform functionalization of Ge, while XPS analysis demonstrated the effective removal of the native oxide. The effective immobilization of the protein was verified by changes in the fluorescence intensity observed through fluorescence microscopy.

To summarize, thiolation is a reliable method for creating stable SAMs on Ge surfaces, forming strong Ge-S bonds that enhance resistance to oxidation, due to the good packing of the chains constituting the SAM, with only few sites exposed to oxidation. Longer alkanethiol chains provide additional protection by improving monolayer packing density and stability through van der Waals interactions, which increase with chain length. This increased hydrophobicity and structural alignment contribute to effective passivation, making thiolation a valuable approach for extending the longevity and functionality of Ge in technological applications.

### 4.4. Arylation

Recently, the use of diazonium salts for functionalization has emerged as a promising approach [45]. Grafting via an electrochemical approach is a well-established technique for Si functionalization [53,55,78,79,80], particularly when employing diazonium salt-based molecules [55]. However, this procedure is not commonly applied to Ge substrates, where only electrochemical etching methods have been reported [55,81]. Notably, this functionalization technique does not yield results on Ge comparable to those obtained on Si [81]. For these reasons, the grafting of diazonium salts on Ge substrates is predominantly performed via wet chemical techniques. This technique allows for the rapid and efficient grafting of organic layers onto Ge surfaces at room temperature, providing a robust passivation strategy that can be tailored for specific applications.

**Figure 7:**
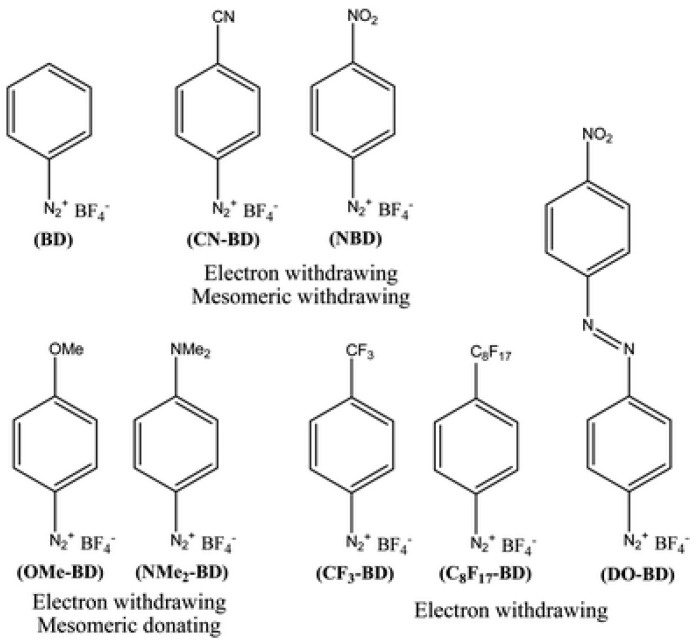
Examples of Diazonium salts used for Ge functionalization and the electronic effects of groups in para-position compared to benzenediazonium. Reproduced with permission from [45] by The Royal Society of Chemistry.

Arylation of Ge has become a significant area of research due to its implications for enhancing the functionality of Ge surfaces in various applications. The process of arylation typically involves the introduction of aryl groups onto Ge surfaces, which can be achieved through several methodologies, including the use of arenediazonium salts and other arylating agents. Examples of molecules used in arylation of Ge are reported in Figure 7.

The process of synthesizing diazonium salts from amines is well known, and these salts can be easily attached to various surfaces through chemical or electrochemical reduction or, in some cases, through spontaneous grafting. Spontaneous grafting has been observed on carbon, metals, and semiconductors such as silicon (Si) and gallium arsenide (GaAs) [45].

**Figure 8:**
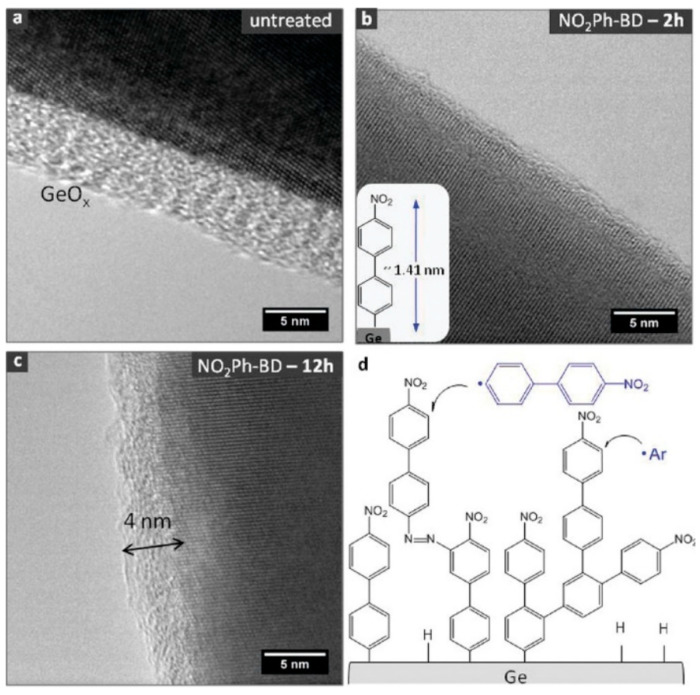
TEM images of (**a**) nonfunctionalized Ge nanowire, (**b**) Ge nanowire functionalized with NO_2_Ph—BD after 2 h reaction time, (**c**) NO_2_Ph—BD functionalized Ge nanowire after 12 h reaction time, and (**d**) schematic illustrating the formation of aryl multilayers and possible introduction of azo species during multilayer formation. Reproduced with permission from [46] by American Chemical Society.

The foundational studies in this field date back to the work of Collins et al. (2011), who investigated the functionalization of Ge NWs s through the use of arenediazonium salts [46]. The nanowires were immersed in freshly prepared diazonium salt solutions (1–5 mM) in anhydrous deoxygenated acetonitrile (MeCN). Functionalization reactions were carried out at room temperature in a glovebox, or at 50 °C on a Schlenk line under a N_2_ atmosphere. This method involves grafting aryl groups onto H-terminated Ge NWs, which were immersed in aqueous HF prior to functionalization. Functionalization reactions were conducted at 50 °C and room temperature to explore the possibility of a spontaneous reaction with the H—Ge surface. Two different reaction pathways were proposed, one based on the formation of an aryl radical and the other proceeding through the formation of an aryl cation. While a radical mechanism similar to that observed for Si surfaces may be responsible for the spontaneous grafting to Ge NWs, Collins and coworkers showed that thermal initiation with heating of the diazonium salts clearly plays a role in the functionalization process. The successful functionalization of the NWs has been confirmed through XPS, ATR-FTIR, and TEM analyses. The TEM image reported in Figure 8a depicts untreated, oxidized Ge NWs with a typical oxide thickness of 2–4 nm. In Figure 8b,c, NWs had undergone functionalization by immersion into nitrobiphenyldiazonium tetrafluoroborate (NO_2_Ph—BD) solutions for 2 h and 12 h, respectively. When the reaction time was 2 h, a thin (1.5–2 nm) homogeneous organic layer formed on the NWs surface. After 12 h, the thickness of the organic film increased to approximately 4 nm, accompanied by a slight decrease in uniformity relative to the thinner functionalization layer. TEM analysis suggests the presence of aryl multilayers, which form as a result of an aryl radical attaching to an already covalently bound ligand, as illustrated in Figure 8d.

In 2012, Lefevre et al. [45] explored the use of diazonium salts for functionalizing Ge flat surfaces. They observed the spontaneous grafting of several different diazonium salts onto Cl-terminated Ge surfaces at room temperature and with very short reaction times. After preparation of the halide-terminated surface, the samples were dipped in a degassed 5 × 10^−3^ M solution of diazonium salt in MeCN. The salts were spontaneously reduced, even at room temperature, forming organic films on the Ge surfaces, as confirmed by XPS analysis. AFM and XPS studies demonstrated that the surface state and the thickness of the layer were influenced by the temperature, reaction time, and electronic effects of the substituents present on the specific diazonium salt. The XPS analysis determined the absolute coverage, which was comparable to the coverage obtained by SAM functionalization (around 50%). This approach allowed for the rapid grafting of organic moieties onto Ge surfaces, demonstrating that the functionalization could be achieved in less than 30 min under mild conditions.

However, the stability of the resulting arylated layers can be variable, and the presence of steric hindrance from bulky substituents may limit the effectiveness of the reaction. Furthermore, the formation of multilayers can complicate the uniformity of the functionalization, as observed in the studies by Collins et al. [46], where the thickness of the functionalization layer was influenced by the substituents on the aromatic ring.

## 5. Conclusions and Perspectives

The functionalization of germanium surfaces represents a pivotal advancement in both semiconductor technology and biotechnological applications. While initial efforts primarily focused on surface passivation and stabilization—addressing the challenges posed by Ge’s unstable oxide layer—recent developments have extended the scope of functionalization, allowing precise modulation of Ge’s chemical, electronic, and optical properties. These advancements open new opportunities in fields such as biosensing, bioimaging, and nanotechnology, where Ge’s high carrier mobility and biocompatibility can be effectively leveraged for enhanced performance.

Nowadays, the applications of germanium functionalized surfaces are predominantly focused on the immobilization of biomolecules for sensing purposes [22,23,57,74,82,83]. However, the functionalization techniques are not yet fully established to enable widespread practical applications. In several cases, the research is limited to theoretical models that hypothesize how the functionalization of these surfaces with organic molecules could be achieved and utilized [1,84,85,86]. Further experimental validation and optimization of these approaches are required to bridge the gap between theoretical predictions and real-world implementations.

It is important to note that germanium wet chemistry is a relatively new area of research and has revealed significant differences compared to well-established silicon chemistry. These differences, including the distinctive reactivity of Ge surfaces depending on their surface orientation and exposure to oxygen, underscore the need for further efforts to refine and optimize these methods. Although the current functionalization techniques are promising, challenges remain, particularly regarding surface reoxidation, long-term stability, and the degree of coverage—parameters that are often evaluated indirectly through techniques such as contact angle measurements rather than direct characterization.

Among the functionalization techniques explored, wet chemical methods such as Grignard reactions, hydrogermylation, thiolation, and diazonium salt grafting have been instrumental in advancing the understanding of Ge surface chemistry. The use of citric acid has also emerged as a notable green and effective passivation strategy, minimizing environmental impact while achieving surface stabilization. However, further studies are required to address the variability of results and to standardize reaction conditions such as pH, temperature, and reagent concentration to ensure reproducibility.

We emphasize that advancements in wet chemistry techniques must be accompanied by the development of appropriate characterization methods. Techniques capable of providing precise and direct assessments of surface modifications will be critical in elucidating the reactivity of Ge surfaces, particularly in relation to surface indexes and the influence of crystal facets. Electrochemical functionalization, for example, is well established for Si but remains underexplored for Ge. Its success will likely depend on combining optimized reaction protocols with robust characterization tools to unlock its full potential.

The growing interest in germanium’s biocompatibility further positions wet chemical functionalization as a promising approach for biomedical applications. Unlike vapor-phase or high-temperature methods, wet techniques offer unique advantages, such as the ability to functionalize large surface areas, accommodate thermally sensitive or high-molecular-weight molecules, and address substrates with complex geometries. These characteristics are particularly valuable in biomedical applications, where delicate molecules such as proteins or polymers must be immobilized without degradation.

Looking forward, significant efforts are still required to overcome current limitations, including optimizing reaction conditions, mitigating toxicity concerns, and improving long-term stability. By refining wet chemical processes and adopting sustainable approaches like citric acid passivation, it will be possible to further unlock the potential of Ge-based materials. Combining advancements in chemistry, characterization, and application-specific development will pave the way for germanium’s integration into next-generation technologies, particularly in biomedicine, electronics, and photonics.

In this review, we have aimed to highlight these critical aspects and emerging challenges while also underscoring the promising opportunities that lie ahead for Ge wet chemistry and functionalization techniques.

## Figures and Tables

**Table 1:** Summary of functionalization methods and characterization reported in this review.

Ge Substrate	Pretreatment	Functionalization Method	Functional Molecules	Characterization Technique	Reference
Ge (111)	HCl	Alkylation	CH_3_(CH_2_)_n_MgX (n = 1, 2, 9, 13, 14, and 17; X = Cl, Br) and R(CH_2_)_2_MgX (R = C_6_H_5_ and CH=CH_2_	ATR-FTIR), (XPS), ellipsometric measurements	[7]
Ge nanowires	HCl, HBr, HI	Alkylation, Thiolation	dodecylmagnesium bromide (DD-MgBr), dodecanethiol	SEM, TEM, ATR-IR, XPS	[8]
Ge (111)	HF	Alkylation	decylmagnesium bromide	SHG, XPS, AFM	[39]
Ge nanowires	HF, HCl	Alkylation, Thiolation	various chain-length alkanethiols and by Grignard reagents (for C2, C8, and C12)	SEM, TEM, XPS, FTIR	[54]
Ge (100)	HF	Hydrogermylation	1-dodecyne, 1-pentyne, 1-dodecene, 1-hexadecene	ATR-FTIR, WCA	[5]
Ge nanowires	HCl, HF	Hydrogermylation, thiolation	alkene, alkyne, or diene species, 1-octanethiol	HRTEM, HRSEM, FTIR, XPS, EDS, EELS,	[71]
Ge (111)	HF	Thiolation	Octanethiol, octadecanethiol	XPS, WCA, AFM, Spectroscopic Ellipsometry, HREEL	[72]
Ge (100) and Ge (111)	HCl, HBr	Thiolation	1-octadecanethiol (ODT)	WCA, ellipsometry, XPS, AES, FTIR	[37]
Ge (100) and Ge (111)	HCl, HBr	Thiolation	1-octadecanethiol (ODT)	SR-PES	[38]
Ge (100) and Ge (111)	-	Thiolation	1-dodecanethiol (C12), 1-octadecanethiol (C18), 11-mercaptoundecanoicacid(MUDA),1-mercaptoundecanol (MUDOL), 3-mercaptoN-nonylpropionamide (1ATC9)	WCA, XPS, AFM, STM	[30]
Ge (100)	Citric acid	Thiolation	dodecanethiol(C12-SH), hexanedithiol (C6-dSH), and butanedithiol (C4-dSH).	FTIR, XPS	[42]
Ge (111)	HCl	Thiolation	1-Dodecanethiol	AFM, ellipsometry, XPS, WCA	[73]
Ge (100)	HCl	Thiolation	1-dodecanethiol	AFM, WCS, XPS	[1]
Ge (100)	HCl	Thiolation	ethanethiol (ET, C2), 1-butanethiol (BT, C4), 1-hexanethiol (HT, C6), 1-octanethiol (OT, C8) and 1-dodecanethiol (DT, C12)	WCA, XPS	[59]
Ge (111)	HCl	Thiolation	11-Mercaptoundecanoic acid (HS(CH2)10COOH) + immobilization of a fluorescent protein	WCA, AFM, XPS, fluorescence microscopy	[74]
Ge (100)	HCl	Arylation	Various synthesized diazonium salts	AFM, XPS	[45]
Ge nanowires	HF	Arylation	Various synthesized diazonium salts	SEM, TEM, XPS	[46]

## Data Availability

No new data were created or analyzed in this study. Data sharing is not applicable to this article.
